# Organ crosstalk and dysfunction in sepsis

**DOI:** 10.1186/s13613-024-01377-0

**Published:** 2024-09-19

**Authors:** André Borges, Luís Bento

**Affiliations:** 1 Intensive Care Unit of Hospital de São José, Unidade de Urgência Médica, Rua José António Serrano, Lisbon, 1150-199 Portugal; 2 NOVA Medical School, Campo dos Mártires da Pátria 130, Lisbon, 1169-056 Portugal

**Keywords:** Organ crosstalk, Sepsis, Organ failure, Multiple organ dysfunction syndrome (MODS)

## Abstract

Sepsis is a dysregulated immune response to an infection that leads to organ dysfunction. Sepsis-associated organ dysfunction involves multiple inflammatory mechanisms and complex metabolic reprogramming of cellular function. These mechanisms cooperate through multiple organs and systems according to a complex set of long-distance communications mediated by cellular pathways, solutes, and neurohormonal actions. In sepsis, the concept of organ crosstalk involves the dysregulation of one system, which triggers compensatory mechanisms in other systems that can induce further damage. Despite the abundance of studies published on ​​organ crosstalk in the last decade, there is a need to formulate a more comprehensive framework involving all organs to create a more detailed picture of sepsis. In this paper, we review the literature published on organ crosstalk in the last 10 years and explore how these relationships affect the progression of organ failure in patients with septic shock. We explored these relationships in terms of the heart–kidney–lung, gut-microbiome–liver–brain, and adipose tissue–muscle–bone crosstalk in sepsis patients. A deep connection exists among these organs based on crosstalk. We also review how multiple therapeutic interventions administered in intensive care units, such as mechanical ventilation, antibiotics, anesthesia, nutrition, and proton pump inhibitors, affect these systems and must be carefully considered when managing septic patients. The progression to multiple organ dysfunction syndrome in sepsis patients is still one of the most frequent causes of death in critically ill patients. A better understanding and monitoring of the mechanics of organ crosstalk will enable the anticipation of organ damage and the development of individualized therapeutic strategies.

**Supplementary Information:**

The online version contains supplementary material available at 10.1186/s13613-024-01377-0.

## Background

What is life? We can characterize life as any entity with the capacity for organization, metabolism, growth, reproduction, environmental adaptation, and response to stimuli [1]. The ability of a biological system to maintain internal balance in the face of external variations is defined as homeostasis. In complex organisms, maintaining homeostasis requires regulating and synchronizing multiple functions of different organs and systems through interorgan communication. Organ crosstalk is the intricate network of long-distance communication between different organs, facilitated by cellular pathways, solutes, neurohormonal actions, and extracellular vesicles (EVs) [2–4].

Sepsis is an uncontrolled immune response to an infection that causes organ dysfunction. Septic shock is a severe form of sepsis with significant circulatory, cellular, and metabolic dysfunction [5]. It is a common condition in intensive care units (ICUs) in which organ failure may progress to multiple organ dysfunction syndrome (MODS) and death [6]. One study reported that the occurrence rates of sepsis in the ICU ranged from 13.6 to 39.3%, with ICU and hospital mortality rates of 25.8% and 35.3%, respectively [7].

Sepsis-associated organ dysfunction involves disruption of organ crosstalk, but the mechanisms underlying this process have not yet been fully elucidated [8]. Formulating a comprehensive framework involving the simultaneous communication mechanisms between all organs is necessary to create a more precise model for predicting organ dysfunction in sepsis patients.

## Literature review of organ crosstalk in sepsis

We carried out a narrative review for articles in PubMed published between 2012 and 2023 with the keywords “organ crosstalk”, “interorgan communication”, “sepsis”, “shock” and “organ failure”. This time frame was chosen because many publications in this area have been published more recently. Original and review articles were included. One major limitation of this review is bias in selecting articles, which may result in the exclusion of significant data.

The selected articles revealed significant work on the brain, respiratory, cardiovascular, renal, hepatic, and hematological systems. There has also been a substantial number of publications on other regulatory systems, particularly the gut-microbiome and adipose–muscle–bone systems, that play crucial roles in regulating metabolism and the inflammatory response to injury.

All systems in the body are deeply integrated, and any classification system is artificial and incompletely describes the full complexity of organ crosstalk. We first reviewed the inflammatory response and metabolic reprogramming in sepsis to facilitate the exposition of concepts. We then explored the impact of organ crosstalk in the following systems: heart-lung-kidney, gut-microbiome-liver-brain, and adipose-muscle-bone. We have categorized these sections based on our experience to enhance the coherence and flow of this review.

### The inflammatory response and metabolic reprograming in sepsis

**Fig. 1:**
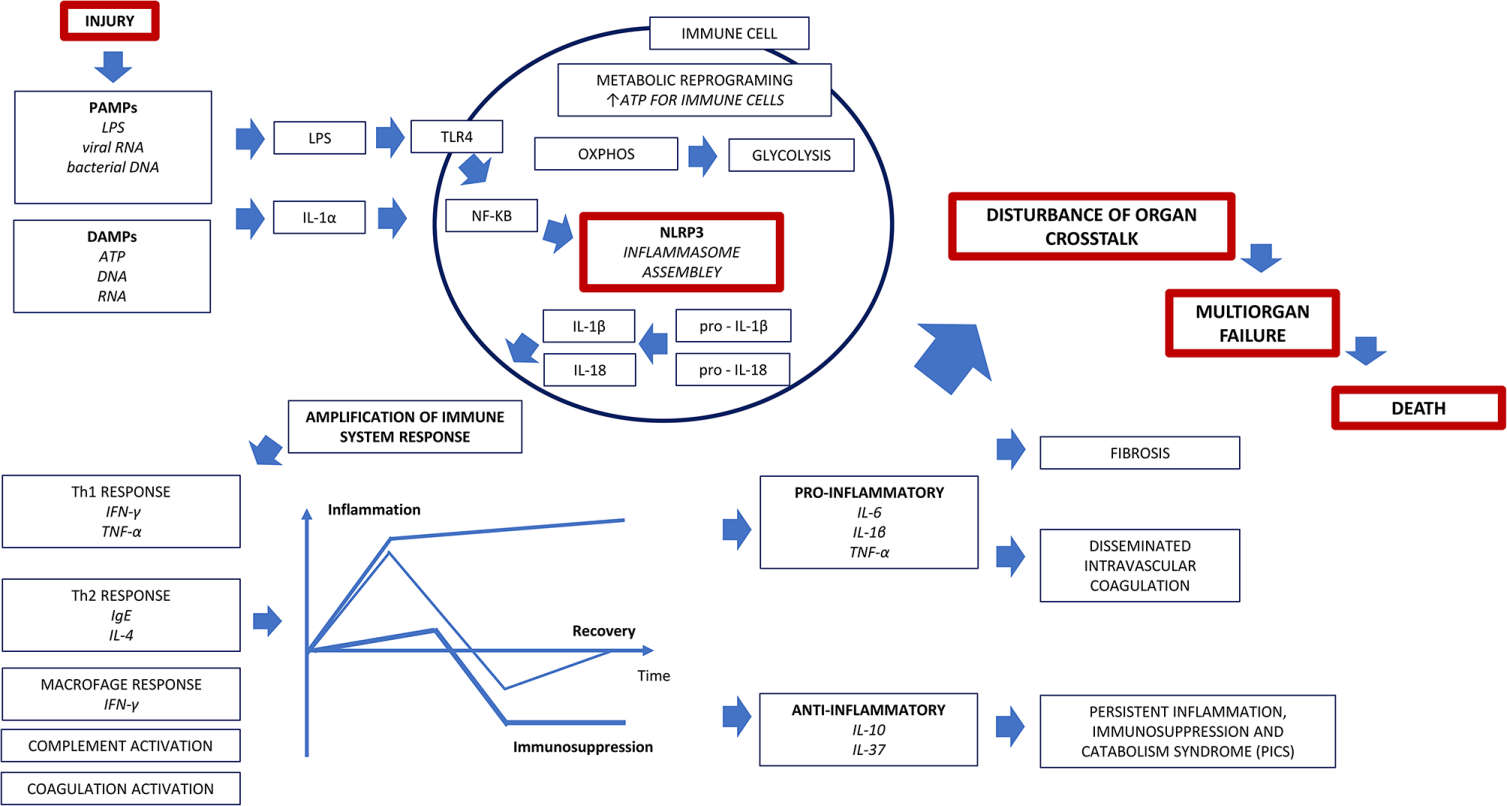
Inflammatory response and metabolic reprogramming in sepsis

The inflammatory response in sepsis begins with an injury or insult (Fig. 1) that, through multiple mechanisms, promotes the activation of inflammasomes in the innate immune system [9]. This results in a robust proinflammatory response, with the release of interleukin-6 (IL-6), tumor necrosis factor-alpha (TNF-α), and interferon-gamma (IFN‐γ) and activation of the coagulation system [10–13].

This immune system activation requires metabolic reprogramming with a shift in metabolism from oxidative phosphorylation (OXPHOS) toward a glycolytic phenotype as the main energy-producing pathway [14]. This results in reduced energy usage that may impact organ cell function, potentially contributing to organ failure and disrupting organ crosstalk [14]. Switching from glycolysis to OXPHOS is essential to restore normal organ function [15].

Adaptive immunity and counterregulatory mechanisms control the intensity and duration of the inflammatory response [16, 17]. Cortisol participates in regulating the balance between hyperinflammation and immunosuppression [18], and patients with inadequate cellular corticosteroid activity due to the severity of the illness develop critical illness-related corticosteroid insufficiency (CIRCI) [19]. In a meta-analysis, it was suggested that corticosteroids may lower mortality rates in sepsis and septic shock [20].

If not regulated, the early hyperinflammatory phase may progress to an overwhelming inflammatory response characterized by refractory shock, MODS, and death [17]. Conversely, patients who survive the early hyperinflammatory phase may progress to a phenotype termed persistent inflammation, immunosuppression, and catabolism syndrome (PICS) [21]. This biphasic view oversimplifies a dynamic process that balances the extremes of hyperinflammation and immunosuppression [22]. The regulation of this delicate balance of the inflammatory response is thought to be strongly influenced by organ crosstalk.

Techniques such as high-volume hemofiltration, plasma adsorption, and hemoadsorption have been designed to target circulating inflammatory molecules in patients with sepsis and multiple organ dysfunction [23]. However, there is a lack of knowledge concerning the interaction between organ crosstalk and artificial organ support systems [23].

In conclusion, sepsis-induced injury triggers a profound inflammatory response by recruiting different systems to counter infection. This response may come at the cost of injury to healthy tissues, which can ultimately compromise organ function. Several regulatory loops regulate the intensity and duration of the hyperinflammatory response so that organs can restore homeostasis. This balance may not be achieved in sepsis with sequential multiorgan failure. Injury to one organ may cause secondary damage or dysfunction in other organs by activating a vicious cycle and worsening MODS [24]. In the following sections, we explore how organ crosstalk is affected in sepsis.

### Heart–lung–kidney crosstalk in sepsis

#### The cardiovascular system

**Fig. 2:**
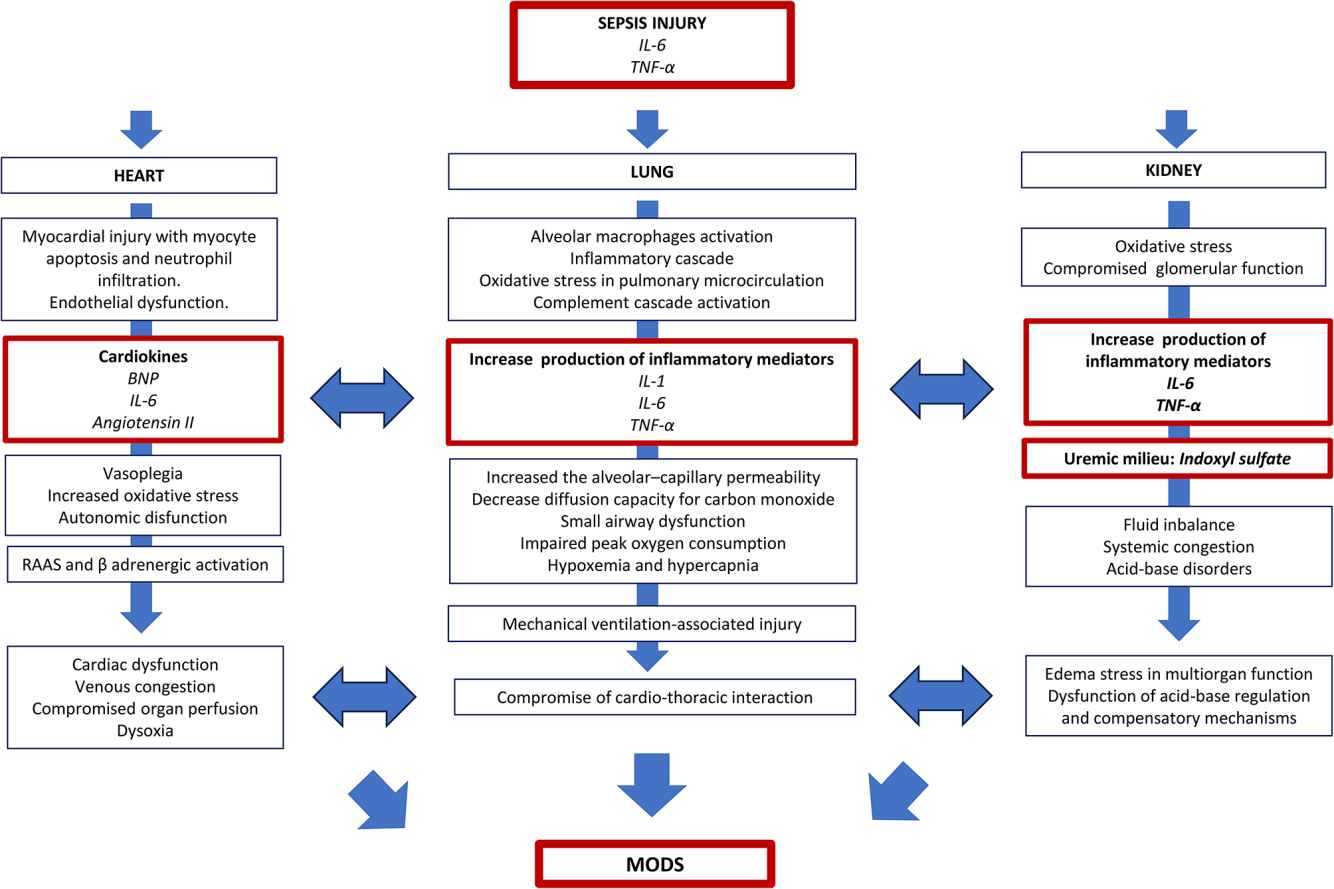
Heart-lung-kidney crosstalk in sepsis

The cardiovascular, respiratory, and renal systems are closely connected and mutually dependent (Fig. 2) [25, 26]. Management of cardiovascular system dysfunction commonly focuses on regulating blood pressure and ensuring proper organ perfusion. While these are a core focus of intervention in ICU, inter-organ communication mechanisms also impact the response to sepsis.

The early hyperinflammatory response affects the endothelial production of nitric oxide, prostacyclin, and inflammatory cytokines [27]. Refractory hypotension follows and is a hallmark of septic shock. Several neurohormonal mechanisms, such as the renin-angiotensin-aldosterone system (RAAS) and ß-adrenergic nervous system, become activated to maintain cardiac output in decompensated cardiovascular function [28]. A growing body of evidence indicates that the heart also acts as a sophisticated paracrine and endocrine organ, synthesizing and secreting proteins called cardiokines, which are involved in intercellular and interorgan communication [29–31]. More than 16 cardiokines have been identified, including atrial natriuretic factor (ANF), brain natriuretic peptide (BNP), transforming growth factor beta-1 (TGF-β1), angiotensin II, and proinflammatory cytokines such as IL-6 [32] which is associated with a decrease in heart rate variability, a hallmark of autonomic dysfunction during sepsis [33]. Cardiokines may significantly regulate communication between the cardiovascular system and other organs during sepsis.

#### The kidney

The establishment of a proinflammatory milieu in sepsis induces acute kidney injury (AKI) [34]. AKI is associated with modulation of the functioning of other vital organs via organ crosstalk [35–42]. The upregulation of TNF-α and IL-6, along with the increase in uremic molecules such as indoxyl sulfate, is important for mediating the effects of AKI on distant organs [37]. This can affect the heart, lungs, central nervous system, hematologic system, liver, gut, and microbiome [43].

AKI is associated with impaired lung function [25, 35, 44]. On the other hand, the development of acute respiratory distress syndrome (ARDS) in sepsis may induce AKI [44]. Biotrauma linked to mechanical ventilation can also result in AKI [45].

In sepsis, the heart and kidney are commonly injured and affect each other through several mechanisms, including organ crosstalk, as exemplified by the cardiorenal syndrome (CRS) type 5 [25, 46]. AKI-induced volume overload, uremic toxin retention, and RAAS overactivation accelerate heart failure [47]. The accumulation of uremic toxins, metabolic acidosis, and electrolyte imbalances leads to cardiovascular toxicity and can increase the risk of myocardial ischemia and life-threatening arrhythmias [39].

Renal replacement therapy (RRT) is frequently utilized to provide essential renal support in treating septic patients in intensive care [48]. RRT effectively aids in controlling blood volume and reducing the concentration of uremic toxins, eventually helping to balance disturbed organ crosstalk, but more studies are needed about the interaction of these techniques and organ crosstalk current.

#### The lung

The development of ARDS, a syndrome of acute respiratory failure due to diffuse lung inflammation and edema not fully explained by cardiac failure or fluid overload [49], is common in sepsis, either as the result of infection or systemic inflammation. The inflammatory cascade initiated in the lungs propagates into circulation and can reach distal organs, thus playing a pivotal role in developing MODS [4, 49, 50].

The central nervous system may also regulate the inflammatory response in the lungs [51, 52]. The vagus nerve is involved in the cholinergic anti-inflammatory pathway (CAP) in the brain–lung axis, in which acetylcholine (ACh) is released and acts on ACh receptors (α7nAChR) on immune cells and pulmonary neuroendocrine cells (PNECs). Proper activation promotes the regression of inflammation, but overreaction may aggravate infection and even promote the occurrence of lung disease [52]. There is some preliminary experimental evidence of CAP in humans. However, this literature has not been well integrated and critically evaluated [53].

Protective mechanical ventilation may be crucial for minimizing lung injury and secondary brain injury [51]. Several sedatives and analgesics may modulate the lung inflammatory response. Morphine inhibits the release of interleukin-17 (IL-17) in the respiratory epithelium, leading to delayed pathogen clearance and sustained inflammation [54]. Dexmedetomidine reduces the inflammatory response to injurious mechanical ventilation by mitigating α2-adrenoceptor activation [55]. Propofol is also known to have neuroimmunomodulatory effects [51].

Extracorporeal techniques such as veno-venous extracorporeal membrane oxygenation (VV-ECMO) are commonly used to provide respiratory support for patients with severe lung disease due to sepsis. Patients on VV-ECMO are at risk for AKI [56], but the impact of these techniques on organ crosstalk is largely unknown.

In conclusion, a pro-inflammatory environment in sepsis triggers a cascade of responses that impact cardiovascular, kidney, and lung functions. The communication between these systems goes beyond organ perfusion, acid-base regulation, and gas exchange. It also involves various metabolites, such as cardiokines, inflammatory mediators, and uremic metabolites like indoxyl sulfate, which can act as carriers of information between organs. The dysregulation of this delicate system disrupts organ function in sepsis and promotes MODS.

### Gut–microbiome–liver–brain crosstalk in sepsis

#### The gut and the microbiome

The digestive system and the gut microbiome form a crucial symbiotic relationship. The gut microbiota serves vital functions, including metabolizing non-digestible components of food, protecting the host from pathogenic invasion, and modulating the immune system [57].

**Fig. 3:**
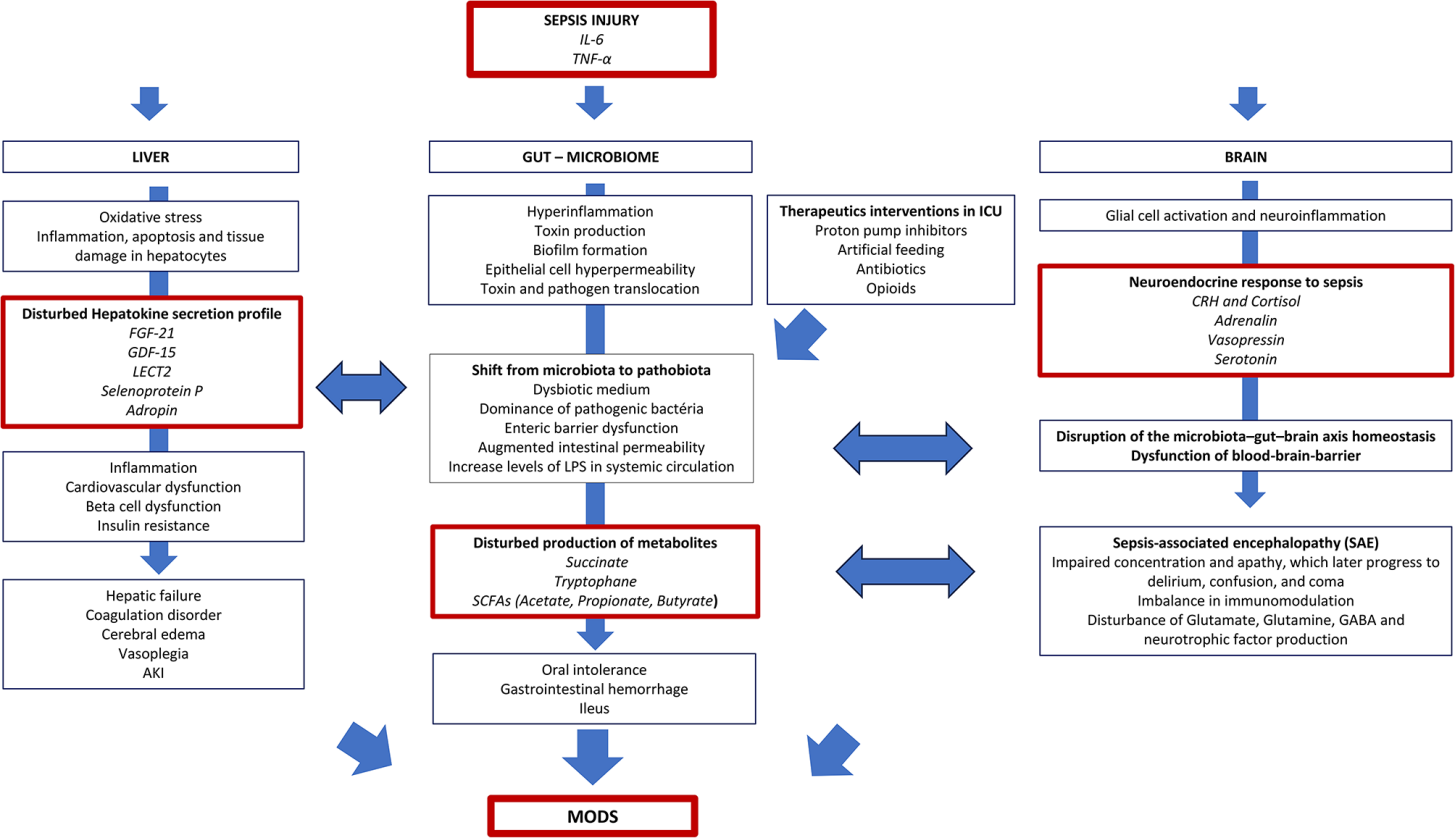
Gut-microbiome-liver-brain crosstalk in sepsis

In septic patients, the protective mechanisms that maintain the gut barrier may fail due to circulatory hypoperfusion, subsequent ischemia, inflammation injury of the enteric barrier, and neuroendocrine dysregulation (Fig. 3) [58, 59]. Sepsis-induced cytokines, such as IL-6, TNF-α, and interleukin-1β (IL-1β), can directly affect the gut barrier by affecting intestinal cell proliferation and apoptosis [60]. Sepsis also alters the symbiotic intestinal microenvironment into a dysbiotic medium that promotes epithelial cell hyperpermeability and apoptosis, hyperinflammation, and the dominance of pathogenic bacteria [61]. This leads to enteric barrier dysfunction, which allows toxins and bacteria to cross into the lymphatic system [60]. The result is gastrointestinal failure, which coincides with clinical signs such as oral intolerance, gastrointestinal hemorrhage, or ileus. It is also associated with lipopolysaccharides (LPS) endotoxemia, directly injuring the liver [60].

The gut microbiome plays a central role in regulating the inflammatory response in sepsis by releasing multiple mediators such as short-chain fatty acids (SCFAs – such as acetate, propionate, and butyrate), succinate, and serotonin, among many others [61–63]. In sepsis, the gut microbiome may be compromised, significantly decreasing microbial diversity, especially anaerobic species, resulting in microbial dysbiosis, which can lead to inadequate immune functioning and inflammatory responses, affecting organ crosstalk resulting in other organ dysfunction [57, 64, 65]. There is a strong connection between the gut microbiome and liver dysfunction in sepsis [66].

The shift from a healthy microbiome to a pathobiome in septic patients may also be driven by antibiotics and intensive care-specific treatments such as artificial feeding, mechanical ventilation, proton pump inhibitors (PPIs), vasopressors, and opioids [59]. Most ICU patients receive antibiotics, which deplete commensal gut bacteria, enrich opportunistic pathogens, and disturb the immune response and physiological activity, influencing other organ functions [61]. Even with short-term antibiotic administration, gut microbiome perturbation can persist for months [66]. The frequent use of PPIs in hospitalized patients is associated with decreased bacterial richness and profound changes in the gut microbiome. One study revealed that 20% of the identified bacteria showed significant deviations, with the abundance of oral bacteria and potential pathogenic bacteria increasing in the gut microbiota of PPI users [67].

#### The liver

The liver plays a crucial role in metabolism, immunity, digestion, detoxification, and vitamin storage. In sepsis, the liver may be damaged by pathogens, toxins, or inflammatory mediators that induce oxidative stress, damaging hepatocytes and resulting in liver dysfunction with severe disruption of organ crosstalk [68]. Liver failure in sepsis is clinically characterized by shock, jaundice, coagulopathy, AKI, hypoglycemia, and brain edema [69].

The liver releases multiple inflammatory factors, such as TNF-α, triggering various local and systemic immune responses [70]. It also produces several endocrine-like hepatokines that play critical roles in regulating extrahepatic metabolism, such as adropin, fibroblast growth factor-21 (FGF-21), hepassocin, leukocyte cell-derived chemotaxin‐2 (LECT2), and selenoprotein P, among others [71–77].

The dysregulation of liver immune function and hepatokine secretion profile must deeply affect organ crosstalk in sepsis, leading, among others, to cardiovascular dysfunction, inflammation, and insulin resistance [73]. These effects may exacerbate subsequent organ injury and lead to progression to MODS in sepsis.

The liver and kidney play crucial roles in maintaining body homeostasis and eliminating metabolic byproducts and drugs, highlighting a deep interconnection between the two organs. Dysfunction of one of these organs in sepsis can promote significant dysfunction in another [42, 78–80].

There are extracorporeal techniques aimed at providing hepatic support in patients with liver failure, such as MARS^®^ and Prometheus^®^, through the clearance of inflammatory mediators and bilirubin. One study demonstrates that MARS^®^ and Prometheus^®^ could clear cytokines from plasma but did not significantly change serum cytokine levels [81].

#### The brain

Sepsis patients commonly develop brain dysfunction [82, 83]. Early clinical signs include clumsiness, fatigue, impaired concentration, and apathy, which may later progress to delirium, confusion, and coma.

The gut microbiome and the liver deeply affect brain function in septic patients [57, 84–89]. The gut microbiome’s production of SCFAs influences the production of neurotransmitters such as glutamate, glutamine, and γ-aminobutyric acid (GABA), among others [90]. 95% of serotonin is produced from tryptophan produced in the gut by the microbiome [91, 92]. Serotonin is an important neurotransmitter that regulates behavior and memory.

Sepsis disrupts microbiota–gut–brain axis homeostasis, thereby causing neurological dysfunction with impairments in memory, concentration, verbal fluency, and executive functioning [89], possibly resulting in sepsis-associated encephalopathy (SAE). Whether systemic infection affects the intestinal microbiota that induces SAE or whether SAE is caused exclusively by a dysregulated host immune response remains unclear [89]. Some of the late features of infection-induced sickness are comparable to the clinical symptoms of depression. Severe sepsis can be associated with brainstem dysfunction, which is clinically characterized by impaired heart rate variability with decreased sympathovagal balance and respiratory rate variability [83, 93]. The brain dysfunction in sepsis is also linked to the disruption of the circadian clock, which plays a crucial role in regulating immune functions and inflammatory responses [94].

In conclusion, the gut microbiome plays a central role in regulating the inflammatory response. In sepsis, there is a shift from a healthy microbiome to a pathobiome, which can lead to gastrointestinal failure. This failure can, in turn, induce dysfunction in other organs through organ crosstalk. Multiple therapeutic interventions in the ICU, such as antibiotics, anesthesia, nutrition, and PPIs, affect the gut microbiome. The liver plays a crucial role in maintaining body homeostasis by eliminating metabolic byproducts and regulating immunity, releasing multiple inflammatory factors that broadly participate in organ crosstalk affecting numerous organs. The dysregulation of liver function deeply affects organ crosstalk in sepsis, leading to cardiovascular dysfunction and inflammation, ultimately resulting in MODS. The gut microbiome and the liver have a close, bidirectional interaction with the brain. Sepsis disrupts the homeostasis of the gut-microbiome-liver-brain axis, triggering cognitive impairment and the development of SAE.

### Adipose tissue–muscle–bone crosstalk in sepsis

#### The adipose tissue

Adipose tissue produces and secretes many mediators, collectively called adipokines [30, 71, 76, 77, 95–101]. Adipokines modulate the metabolism of distant organs and tissues such as the liver, pancreas, bone, muscle, and heart. The most well-known adipokines are leptin, adiponectin, resistin, and TNF-α [102]. Leptin acts as a proinflammatory cytokine [102]. Leptin deficiency and leptin resistance induce alterations in cytokine production and increase susceptibility to infectious diseases [103].

Brown adipose tissue (BAT) is responsible for heat production but also secretes molecules called batokines that mediate the general metabolic activity of the liver, heart, muscle and immune functions [104]. Batokines include FGF-21, IL-6, and exosomal microRNAs (miR-99b) [105]. How these adipokines and batokines participate in the inflammatory response in sepsis remains unclear.

#### The bone and the muscle

Both bone and muscle regulate the utilization, distribution, and delivery of nutrients and other substrates [106]. Bone provides the most significant storage site for calcium and phosphate and promotes the production of mesenchymal stem cells and hematopoiesis. Osteokines derived from bone cells, such as osteocalcin and sclerostin, induce muscle anabolism and catabolism [98, 107–110]. There is a strong connection between other regulatory systems and bone metabolism, such as vitamin D metabolism, which involves kidney–liver crosstalk [111].

Muscle is the largest depot for glucose disposal and the storage of amino acids. Myokines derived from myocytes include IL-6, irisin, myostatin, and FGF-21 [77, 97, 98, 106, 112–116]. Muscle disuse and atrophy result in osteoporosis, a process that also involves IL-6 [117].

In conclusion, adipose tissue, muscle, and bone function as metabolic reservoirs and in inflammatory regulation. As these components constitute most of the body mass, they must play a fundamental role in regulating the inflammatory response and interorgan communication in sepsis. In septic patients, immobilization leads to muscle disuse and atrophy, promoting osteoporosis. IL-6 plays an essential role in this process. Overall, the dysregulation of adipose, muscle, and bone metabolism may lead to the dysregulation of thermogenesis and glucose metabolism and impact the inflammatory response. We lack knowledge of how these systems are effectively regulated in sepsis.

## Forward to the future: monitoring organ cross-talk

Organ crosstalk in sepsis involves a complex signaling network in which various mediators participate in multiple regulatory and counterregulatory pathways between native organs. Understanding the diverse and sometimes conflicting actions of the mediators involved in organ crosstalk is challenging (Table 1). The challenge is compounded by the reality that critically ill patients often necessitate multiple support techniques, resulting in intricate crosstalk between their native organs and artificial organ support. A recent review raises this question, categorizing the crosstalk into four major subgroups: between two or more native organs, between native and artificial organs, between two or more artificial organs, and between multiple native and artificial organs, which is frequently observed in critical patients [118].

**Table 1:** Summary list of contributing factors in organ crosstalk in sepsis

Organ system	Contributing factors	Pathophysiological processes in sepsis
Heart (Cardiokines)	Natriuretic peptides: Atrial natriuretic factor (ANF) and B-type natriuretic peptide (BNP)	Regulation of fluid homeostasis, reduction of systemic vascular resistance, and anti-inflammatory effects.
	Angiotensin II	Potent vasoconstrictor with pro-inflammatory profile.
	Interleukin-6 (IL-6)	Central mediator of pro-inflammatory response in the acute phase response of sepsis.
Lung	Interleukin-1 (IL-1) family	Group of cytokines that regulate the inflammatory responses in sepsis.
	Interferon-gamma (IFN-γ)	Critical for innate and adaptive immunity.
	IL-6	Central mediator of pro-inflammatory response in the acute phase response of sepsis.
Kidney	Uremic molecules: Indoxyl sulfate	Regulate signaling and metabolism in multiple organs through a remote sensing and signaling network.
	Interferon-alpha (IFN-α)	Regulator of antiviral immunity.
	IL-6	Central mediator of pro-inflammatory response in the acute phase response of sepsis.
Gut and Microbiome	Short-chain fatty acids (SCFA): acetate, propionate, butyrate	Regulation of the gut microbiome, intestinal epithelial barrier, and inflammatory response. Modulation of the production of neurotransmitters such as Glutamate and γ-aminobutyric acid (GABA).
	Succinate	Function as a metabokine regulating systemic metabolism.
	Tryptophane	Essential amino acid whose metabolites play critical roles in metabolism regulation and inflammation.
Liver (Hepatokines)	Fibroblast growth factor 21 (FGF-21) and Growth/differentiation factor-15 (GDF-15)	FGF-21 regulates metabolism. GDF-15 is expressed in low concentrations in most organs and upregulated in the liver after injury. It also regulates inflammatory pathways.
	Adropin	Regulation of energy metabolism and insulin resistance.
	Selenoprotein P	Regulation of stress responses, metabolism, and immunity with important redox and anti-inflammatory activity.
	Leukocyte cell-derived chemotaxin-2 (LECT2)	Modulate multiple physiological processes, including immune response and glucose metabolism.
	Tumor Necrosis Factor-alpha (TNF-α)	Primary mediator regulating the pro-inflammatory response in sepsis.
Nervous system	Adrenomedullary hormones (catecholamines): Adrenaline, Noradrenaline, and Dopamine.	Regulation of blood pressure and myocardial contractility. Modulation of metabolic responses to stress.
	Hypothalamic hormones: Corticotropin-releasing hormone (CRH)	CRH stimulates the anterior pituitary gland to release the adrenocorticotropic hormone (ACTH), which acts on the adrenal cortex to release Cortisol. Cortisol regulates the stress response and metabolism to injury.
	Neurohypophysial hormones: Vasopressin	Regulation of blood pressure, sodium homeostasis, and kidney function.
	Enteric neurohormones: Serotonin.	Essential functions in learning, and memory. Regulates vasoconstriction, hemostasis, and blood clotting.
Adipose (Adipokines)	Leptin	Pro-inflammatory mediator. Essential in energy metabolism and endocrine modulation.
	Adiponectin	Anti-inflammatory activities and suppression of tumor necrosis factor-alpha (TNF-α). Induces the anti-inflammatory cytokine interleukin-10 (IL-10).
	TNF-α	TNF-α is also produced in adipocytes as an Adipokine, promoting insulin resistance.
Muscle (Myokines)	IL-6	IL-6 produced from muscle in response to muscle contraction is associated with anti-inflammatory functions. The cytokine response to exercise is not preceded by an increase in plasma-TNF-α as in sepsis.
	Myostatin	Myostatin inhibits muscle differentiation and causes muscle atrophy.
Bone (Osteokines)	Osteocalcin	Metabolism regulation.
	Sclerosin	Inhibits muscle formation and hinders bone formation.

Currently, the approach to treating septic shock is based on regular evaluation of the clinical course and treatment efficacy through repeated measurements of metabolites that could be used as biomarkers. This approach has been insufficient for mitigating the mortality associated with organ dysfunction in patients with septic shock. We need to improve our analysis of the impaired organ crosstalk that precedes organ dysfunction.

How can organ crosstalk be tracked in sepsis patients? Metabolomics may be performed in this situation [119]. The applicability of metabolomics studies in clinical practice may provide a better understanding of disease mechanisms and the possibility of developing new diagnostic and therapeutic methodologies. Exploring the metabolic profile that may reflect organ crosstalk is essential for a comprehensive understanding of how organ crosstalk functions [120].

However, the application of metabolomics does not resolve one major limitation. The challenges of causal inference and directionality will remain. It is essential to consider organ crosstalk as a complex language system to address this issue. Language is a system of conventional symbols through which group members express themselves. Some characteristics of effective language include that it is concrete and precise. We know the actions of some mediators in organ crosstalk, but we must obtain a better understanding of the rules that enable concrete and accurate instructions to be constructed. How are those rules defined in a complex system? The relationship between the complexity of a system and the emergence of higher-level properties from the interactions of its components is intriguing and a significant focus in computational analysis [121, 122]. To predict the outcome of such complex systems, like organ crosstalk, it is more important to characterize their functional architecture, in other words, to identify the relevant macroscopic levels that determine the overall result, than to have an extensive microscopic description [123]. Perhaps it is not essential who produces what, but rather how the mediators interact in the relevant macroscopic levels, from which higher-level properties emerge, enabling clarification, for example, of the various sepsis phenotypes [124]. Further work is needed, and perhaps by integrating multiple data sources, including metabolomics profiles, through data fusion, we can develop a more accurate personalized model of the mechanics of sepsis [125] with the potential to create new models of prediction of the evolution of disease and new therapeutics targets [126].

## Conclusions

MODS remains a frequent cause of mortality in septic patients despite the advances in intensive care medicine [23]. Understanding the mechanics of organ crosstalk in sepsis and all the complex signaling mechanisms between organs will enable the anticipation of organ damage and the development of individualized therapeutic strategies for critically ill patients. This review reinforces the intimate and complex connections between multiple organs and systems involved in organ crosstalk in sepsis. New methods are necessary to enable more precise monitoring and the development of individualized therapeutic strategies. The potential application of metabolomics in evaluating organ crosstalk in sepsis is exciting.

## Electronic supplementary material

Below is the link to the electronic supplementary material.

Supplementary Material 1

## Data Availability

Not applicable.

## Abbreviations

Ach: Acetylcholine
AKI: Acute Kidney Injury
ANF: Atrial Natriuretic Factor
ARDS: Acute Respiratory Distress Syndrome
BAT: Brown Adipose Tissue
BNP: Brain Natriuretic Peptide
CIRCI: Critical Illness-Related Corticosteroid Insufficiency
CKD: Chronic Kidney Disease
CRS: Cardiorenal Syndrome
EV: Extracellular Vesicles
FGF-21: Fibroblast Growth Factor 21
GABA: γ-Aminobutyric Acid
HPA axis: Hypothalamic–Pituitary–Adrenal Axis
ICU: Intensive Care Unit
IFN-γ: Interferon‐Gamma
IL-6: Interleukin-6
IL-17: Interleukin-17
IL-1β: Interleukin-1β
LECT2: Leukocyte Cell-Derived Chemotaxin‐2
LPS: Lipopolysaccharide
microRNA: Microribonucleic Acid
MODS: Multiple Organ Dysfunction Syndrome
OXPHOS: Oxidative Phosphorylation
PICS: Persistent Inflammation, Immunosuppression, And Catabolism Syndrome
PNECs: Pulmonary Neuroendocrine Cells
PPIs: Proton Pump Inhibitors
RAAS: Renin-Angiotensin-Aldosterone System
RNA: Ribonucleic Acid
RRT: Renal Replacement Therapy
SAE: Sepsis-Associated Encephalopathy
SCFAs: Short-Chain Fatty Acids
TGF-β1: Transforming Growth Factor-β1
TNF-α: Tumor Necrosis Factor Alpha
VV-ECMO: Venous-Venous Extracorporeal Membrane Oxygenation
