# Supplementary material for: Organ crosstalk and dysfunction in sepsis
Source: Ann Intensive Care. 2024 Sep 19;14:147. doi: 10.1186/s13613-024-01377-0 (PMC11413314; doi:10.1186/s13613-024-01377-0)
Supplement: Supplementary file 1 — Supplementary Material 1 [file 13613_2024_1377_MOESM1_ESM.docx]

**Table 1.** Literature review on organ crosstalk

| Organs | Lungs | Cardiovascular system | Kidneys | Liver | Gut | Adipose tissue | Microbiome | Immune system | Endocrine system | Bone | Muscle |
| --- | --- | --- | --- | --- | --- | --- | --- | --- | --- | --- | --- |
| Brain | [1-4] | [5-7] | [8-10] | [11] | [12-19] | [5, 19-22] | [13-15, 19, 23, 24] | [17, 25-32] | [32-37] | [38-40] | [41-45] |
| Lungs |  | [46, 47] | [8, 46, 48-57] | [58] | [59, 60] | [61, 62] | [4, 59, 63, 64] | [64-70] | [71] | [72] | [73] |
| Cardiovascular system |  |  | [46, 51, 74-91] | [92-96] | [97] | [5, 95, 98-103] | [23, 104] | [94, 105-107] | [108] | [94, 109] | [93, 94, 101, 110-116] |
| Kidneys |  |  |  | [50, 51, 55, 117-123] | [50, 51, 117, 124] | [125, 126] | [104, 127] | [56, 77, 86, 119, 128-132] | [133] | [109] | [134, 135] |
| Liver |  |  |  |  | [136-144] | [93, 94, 102, 140, 145-153] | [104, 138, 139, 154] | [105, 119, 149, 150, 155-157] | [158] | [94, 111] | [93, 94, 111, 115, 151, 152, 159-163] |
| Gut |  |  |  |  |  | [19, 20, 164-169] | [13, 14, 19, 23, 59, 104, 124, 137-139, 154, 164, 166, 167, 170-180] | [17, 23, 136, 167, 171, 172, 178, 181-185] | [177, 179] | [186] | [164, 187, 188] |
| Adipose tissue |  |  |  |  |  |  | [19, 164, 166, 167] | [21, 62, 115, 126, 145, 147, 189-191] | [192] | [94, 125, 193, 194] | [93, 94, 114, 146, 151, 160, 193, 195-200] |
| Microbiome |  |  |  |  |  |  |  | [178, 179, 201] | [202] | [164] | [164, 167] |
| Immune system |  |  |  |  |  |  |  |  | [108] | [94, 193] | [41, 94, 152, 203-205] |
| Endocrine system |  |  |  |  |  |  |  |  |  | [72, 125, 194, 206] | [43, 93, 112, 114, 151, 195, 199, 206-209] |
| Bone |  |  |  |  |  |  |  |  |  |  | [94, 111, 193, 206, 210-217] |

1. Quilez ME, Lopez-Aguilar J, Blanch L. Organ crosstalk during acute lung injury, acute respiratory distress syndrome, and mechanical ventilation. Curr Opin Crit Care. 2012;18:23-8.

2. Li C, Chen W, Lin F, Li W, Wang P, Liao G, et al. Functional Two-Way Crosstalk Between Brain and Lung: The Brain-Lung Axis. Cell Mol Neurobiol. 2023;43:991-1003.

3. Albaiceta GM, Brochard L, Dos Santos CC, Fernandez R, Georgopoulos D, Girard T, et al. The central nervous system during lung injury and mechanical ventilation: a narrative review. Br J Anaesth. 2021;127:648-59.

4. Chen J, Li T, Ye C, Zhong J, Huang JD, Ke Y, et al. The lung microbiome: a new frontier for lung and brain disease. Int J Mol Sci. 2023;24:2170.

5. Czerwinska M, Czarzasta K, Cudnoch-Jedrzejewska A. New Peptides as Potential Players in the Crosstalk Between the Brain and Obesity, Metabolic and Cardiovascular Diseases. Front Physiol. 2021;12:692642.

6. Mohanta SK, Yin C, Weber C, Habenicht AJR. Neuroimmune cardiovascular interfaces in atherosclerosis. Front Cell Dev Biol. 2023;11:1117368.

7. Bekala A, Plotek W, Siwicka-Gieroba D, Solek-Pastuszka J, Bohatyrewicz R, Biernawska J, et al. Melatonin and the brain-heart crosstalk in neurocritically ill patients-from molecular action to clinical practice. Int J Mol Sci. 2022;23:7094.

8. Onal EM, Sag AA, Sal O, Yerlikaya A, Afsar B, Kanbay M. Erythropoietin mediates brain-vascular-kidney crosstalk and may be a treatment target for pulmonary and resistant essential hypertension. Clin Exp Hypertens. 2017;39:197-209.

9. Afsar B, Sag AA, Yalcin CE, Kaya E, Siriopol D, Goldsmith D, et al. Brain-kidney cross-talk: Definition and emerging evidence. Eur J Intern Med. 2016;36:7-12.

10. Li X, Yuan F, Zhou L. Organ Crosstalk in Acute Kidney Injury: Evidence and Mechanisms. J Clin Med. 2022;11.

11. Matsubara Y, Kiyohara H, Teratani T, Mikami Y, Kanai T. Organ and brain crosstalk: The liver-brain axis in gastrointestinal, liver, and pancreatic diseases. Neuropharmacology. 2022;205:108915.

12. Giridharan VV, Generoso JS, Lence L, Candiotto G, Streck E, Petronilho F, et al. A crosstalk between gut and brain in sepsis-induced cognitive decline. J Neuroinflammation. 2022;19:114.

13. Ceppa FA, Izzo L, Sardelli L, Raimondi I, Tunesi M, Albani D, et al. Human Gut-Microbiota Interaction in Neurodegenerative Disorders and Current Engineered Tools for Its Modeling. Front Cell Infect Microbiol. 2020;10:297.

14. Choi TY, Choi YP, Koo JW. Mental disorders linked to crosstalk between the gut microbiome and the brain. Exp Neurobiol. 2020;29:403-16.

15. Fried S, Wemelle E, Cani PD, Knauf C. Interactions between the microbiota and enteric nervous system during gut-brain disorders. Neuropharmacology. 2021;197:108721.

16. Guo C, Huo YJ, Li Y, Han Y, Zhou D. Gut-brain axis: focus on gut metabolites short-chain fatty acids. World J Clin Cases. 2022;10:1754-63.

17. Holzer P, Farzi A, Hassan AM, Zenz G, Jacan A, Reichmann F. Visceral Inflammation and Immune Activation Stress the Brain. Front Immunol. 2017;8:1613.

18. Maiuolo J, Gliozzi M, Musolino V, Carresi C, Scarano F, Nucera S, et al. The Contribution of Gut Microbiota-Brain Axis in the Development of Brain Disorders. Front Neurosci. 2021;15:616883.

19. Yu Z, Wang Y, Yu Z, Lu M, Xu B. Crosstalk between adipose tissue and the microbiota-gut-brain axis in metabolic diseases. Int J Biol Sci. 2022;18:1706-23.

20. Till A, Fries C, Fenske WK. Brain-to-BAT - and back? Crosstalk between the central nervous system and thermogenic adipose tissue in development and therapy of obesity. Brain Sci. 2022;12:1646.

21. Stranahan AM. Visceral adiposity, inflammation, and hippocampal function in obesity. Neuropharmacology. 2022;205:108920.

22. Yerrapragada SM, Bihl JC. Role of Exosomes in Mediating the Cross-Talk Between Adipose Tissue and the Brain. Neuromolecular Med. 2022;24:57-61.

23. Schroeder BO, Backhed F. Signals from the gut microbiota to distant organs in physiology and disease. Nat Med. 2016;22:1079-89.

24. Choi TY, Choi YP, Koo JW. Mental Disorders Linked to Crosstalk between The Gut Microbiome and The Brain. Exp Neurobiol. 2020;29:403-416.

25. Bourhy L, Mazeraud A, Bozza FA, Turc G, Lledo PM, Sharshar T. Neuro-Inflammatory Response and Brain-Peripheral Crosstalk in Sepsis and Stroke. Front Immunol. 2022;13:834649.

26. Choi YH, Laaker C, Hsu M, Cismaru P, Sandor M, Fabry Z. Molecular mechanisms of neuroimmune crosstalk in the pathogenesis of stroke. Int J Mol Sci. 2021;22:9486.

27. Han D, Liu H, Gao Y, Feng J. Targeting brain-spleen crosstalk after stroke: new insights into stroke pathology and treatment. Curr Neuropharmacol. 2021;19:1590-605.

28. Limanaqi F, Biagioni F, Gaglione A, Busceti CL, Fornai F. A Sentinel in the Crosstalk Between the Nervous and Immune System: The (Immuno)-Proteasome. Front Immunol. 2019;10:628.

29. Matejuk A, Vandenbark AA, Offner H. Cross-Talk of the CNS With Immune Cells and Functions in Health and Disease. Front Neurol. 2021;12:672455.

30. Saand AR, Yu F, Chen J, Chou SH. Systemic inflammation in hemorrhagic strokes - a novel neurological sign and therapeutic target? J Cereb Blood Flow Metab. 2019;39:959-88.

31. Yu H, Cai Y, Zhong A, Zhang Y, Zhang J, Xu S. The "Dialogue" Between Central and Peripheral Immunity After Ischemic Stroke: Focus on Spleen. Front Immunol. 2021;12:792522.

32. Goncalves RA, De Felice FG. The crosstalk between brain and periphery: Implications for brain health and disease. Neuropharmacology. 2021;197:108728.

33. Roh E, Song DK, Kim MS. Emerging role of the brain in the homeostatic regulation of energy and glucose metabolism. Exp Mol Med. 2016;48:e216.

34. Arango-Lievano M, Jeanneteau F. Timing and crosstalk of glucocorticoid signaling with cytokines, neurotransmitters and growth factors. Pharmacol Res. 2016;113:1-17.

35. Berthoud HR, Neuhuber WL. Vagal mechanisms as neuromodulatory targets for the treatment of metabolic disease. Ann N Y Acad Sci. 2019;1454:42-55.

36. Gopalakrishnan S, Kannan NN. Only time will tell: the interplay between circadian clock and metabolism. Chronobiol Int. 2021;38:149-67.

37. Hemmati F, Ghasemi R, Ibrahim NM, Dargahi L, Mohamed Z, Raymond AA, et al. Crosstalk between insulin and toll-like receptor signaling pathways in the central nervous system. Mol Neurobiol. 2014;50:797-810.

38. Kelly RR, Sidles SJ, LaRue AC. Effects of Neurological Disorders on Bone Health. Front Psychol. 2020;11:612366.

39. Otto E, Knapstein PR, Jahn D, Appelt J, Frosch KH, Tsitsilonis S, et al. Crosstalk of brain and bone-clinical observations and their molecular bases. Int J Mol Sci. 2020;21:4946.

40. Rousseaud A, Moriceau S, Ramos-Brossier M, Oury F. Bone-brain crosstalk and potential associated diseases. Horm Mol Biol Clin Investig. 2016;28:69-83.

41. Bay ML, Pedersen BK. Muscle-Organ Crosstalk: Focus on Immunometabolism. Front Physiol. 2020;11:567881.

42. Burtscher J, Millet GP, Place N, Kayser B, Zanou N. The muscle-brain axis and neurodegenerative diseases: the key role of mitochondria in exercise-induced neuroprotection. Int J Mol Sci. 2021;22:6479.

43. Delezie J, Handschin C. Endocrine Crosstalk Between Skeletal Muscle and the Brain. Front Neurol. 2018;9:698.

44. Jena BP, Larsson L, Gatti DL, Ghiran I, Cho WJ. Understanding Brain-Skeletal Muscle Crosstalk Impacting Metabolism and Movement. Discoveries (Craiova). 2022;10:e144.

45. Murphy RM, Watt MJ, Febbraio MA. Metabolic communication during exercise. Nat Metab. 2020;2:805-16.

46. Husain-Syed F, McCullough PA, Birk HW, Renker M, Brocca A, Seeger W, et al. Cardio-Pulmonary-Renal Interactions: A Multidisciplinary Approach. J Am Coll Cardiol. 2015;65:2433-48.

47. Mitaka C, Si MK, Tulafu M, Yu Q, Uchida T, Abe S, et al. Effects of atrial natriuretic peptide on inter-organ crosstalk among the kidney, lung, and heart in a rat model of renal ischemia-reperfusion injury. Intensive Care Med Exp. 2014;2:28.

48. Quaglia M, Fanelli V, Merlotti G, Costamagna A, Deregibus MC, Marengo M, et al. Dual role of extracellular vesicles in sepsis-associated kidney and lung injury. Biomedicines. 2022;10:2448.

49. Alge J, Dolan K, Angelo J, Thadani S, Virk M, Arikan AA. Two to Tango: kidney-lung interaction in acute kidney injury and acute respiratory distress syndrome. Front Pediatr. 2021;9:744110.

50. Li X, Yuan F, Zhou L. Organ crosstalk in acute kidney injury: evidence and mechanisms. J Clin Med. 2022;11:6637.

51. Lee SA, Cozzi M, Bush EL, Rabb H. Distant organ dysfunction in acute kidney injury: a review. Am J Kidney Dis. 2018;72:846-56.

52. Husain-Syed F, Slutsky AS, Ronco C. Lung-kidney cross-talk in the critically ill patient. Am J Respir Crit Care Med. 2016;194:402-14.

53. Domenech P, Perez T, Saldarini A, Uad P, Musso CG. Kidney-lung pathophysiological crosstalk: its characteristics and importance. Int Urol Nephrol. 2017;49:1211-5.

54. Ko GJ, Rabb H, Hassoun HT. Kidney-lung crosstalk in the critically ill patient. Blood Purif. 2009;28:75-83.

55. Li X, Hassoun HT, Santora R, Rabb H. Organ crosstalk: the role of the kidney. Curr Opin Crit Care. 2009;15:481-7.

56. Maeda A, Hayase N, Doi K. Acute Kidney Injury Induces Innate Immune Response and Neutrophil Activation in the Lung. Front Med (Lausanne). 2020;7:565010.

57. Paladino JD, Hotchkiss JR, Rabb H. Acute kidney injury and lung dysfunction: a paradigm for remote organ effects of kidney disease? Microvasc Res. 2009;77:8-12.

58. Arteel GE. Liver-lung axes in alcohol-related liver disease. Clin Mol Hepatol. 2020;26:670-6.

59. Anand S, Mande SS. Diet, Microbiota and Gut-Lung Connection. Front Microbiol. 2018;9:2147.

60. Enaud R, Prevel R, Ciarlo E, Beaufils F, Wieers G, Guery B, et al. The Gut-Lung Axis in Health and Respiratory Diseases: A Place for Inter-Organ and Inter-Kingdom Crosstalks. Front Cell Infect Microbiol. 2020;10:9.

61. Kuvat N, Tanriverdi H, Armutcu F. The relationship between obstructive sleep apnea syndrome and obesity: A new perspective on the pathogenesis in terms of organ crosstalk. Clin Respir J. 2020;14:595-604.

62. Palma G, Sorice GP, Genchi VA, Giordano F, Caccioppoli C, D'Oria R, et al. Adipose tissue inflammation and pulmonary dysfunction in obesity. Int J Mol Sci. 2022;23:7349.

63. Santo CE, Caseiro C, Martins MJ, Monteiro R, Brandao I. Gut microbiota, in the halfway between nutrition and lung function. Nutrients. 2021;13:1716.

64. Gosens R, Hiemstra PS, Adcock IM, Bracke KR, Dickson RP, Hansbro PM, et al. Host-microbe cross-talk in the lung microenvironment: implications for understanding and treating chronic lung disease. Eur Respir J. 2020;56:1902320.

65. Yang Z, Nicholson SE, Cancio TS, Cancio LC, Li Y. Complement as a vital nexus of the pathobiological connectome for acute respiratory distress syndrome: An emerging therapeutic target. Front Immunol. 2023;14:1100461.

66. Bissonnette EY, Lauzon-Joset JF, Debley JS, Ziegler SF. Cross-Talk Between Alveolar Macrophages and Lung Epithelial Cells is Essential to Maintain Lung Homeostasis. Front Immunol. 2020;11:583042.

67. Cheresh P, Kim SJ, Tulasiram S, Kamp DW. Oxidative stress and pulmonary fibrosis. Biochim Biophys Acta. 2013;1832:1028-40.

68. Kim SH, Jang YS. Recent Insights into Cellular Crosstalk in Respiratory and Gastrointestinal Mucosal Immune Systems. Immune Netw. 2020;20:e44.

69. Toyoshima S, Okayama Y. Neuro-allergology: mast cell-nerve cross-talk. Allergol Int. 2022;71:288-93.

70. Zamora R, Korff S, Mi Q, Barclay D, Schimunek L, Zucca R, et al. A computational analysis of dynamic, multi-organ inflammatory crosstalk induced by endotoxin in mice. PLoS Comput Biol. 2018;14:e1006582.

71. Ge P, Luo Y, Okoye CS, Chen H, Liu J, Zhang G, et al. Intestinal barrier damage, systemic inflammatory response syndrome, and acute lung injury: A troublesome trio for acute pancreatitis. Biomed Pharmacother. 2020;132:110770.

72. Cutuli SL, Cascarano L, Tanzarella ES, Lombardi G, Carelli S, Pintaudi G, et al. Vitamin D status and potential therapeutic options in critically ill patients: a narrative review of the clinical evidence. Diagnostics (Basel). 2022;12:2719.

73. Whitson BA, Tan T, Gong N, Zhu H, Ma J. Muscle multiorgan crosstalk with MG53 as a myokine for tissue repair and regeneration. Curr Opin Pharmacol. 2021;59:26-32.

74. Mehta RL, Rabb H, Shaw AD, Singbartl K, Ronco C, McCullough PA, et al. Cardiorenal syndrome type 5: clinical presentation, pathophysiology and management strategies from the eleventh consensus conference of the Acute Dialysis Quality Initiative (ADQI). Contrib Nephrol. 2013;182:174-94.

75. Napoli C, Casamassimi A, Crudele V, Infante T, Abbondanza C. Kidney and heart interactions during cardiorenal syndrome: a molecular and clinical pathogenic framework. Future Cardiol. 2011;7:485-97.

76. Buliga-Finis ON, Ouatu A, Badescu MC, Dima N, Tanase DM, Richter P, et al. Beyond the cardiorenal syndrome: pathophysiological approaches and biomarkers for renal and cardiac crosstalk. Diagnostics (Basel). 2022;12:773.

77. Clementi A, Virzi GM, Battaglia GG, Ronco C. Neurohormonal, endocrine, and immune dysregulation and inflammation in cardiorenal syndrome. Cardiorenal Med. 2019;9:265-73.

78. Di Lullo L, Bellasi A, Russo D, Cozzolino M, Ronco C. Cardiorenal acute kidney injury: epidemiology, presentation, causes, pathophysiology and treatment. Int J Cardiol. 2017;227:143-50.

79. Di Lullo L, Reeves PB, Bellasi A, Ronco C. Cardiorenal Syndrome in Acute Kidney Injury. Semin Nephrol. 2019;39:31-40.

80. Forrester SJ, Booz GW, Sigmund CD, Coffman TM, Kawai T, Rizzo V, et al. Angiotensin II signal transduction: an update on mechanisms of physiology and pathophysiology. Physiol Rev. 2018;98:1627-738.

81. Husain-Syed F, Ricci Z, Brodie D, Vincent JL, Ranieri VM, Slutsky AS, et al. Extracorporeal organ support (ECOS) in critical illness and acute kidney injury: from native to artificial organ crosstalk. Intensive Care Med. 2018;44:1447-59.

82. Kaesler N, Babler A, Floege J, Kramann R. Cardiac remodeling in chronic kidney disease. Toxins (Basel). 2020;12:161.

83. Kingma JG, Simard D, Rouleau JR. Renocardiac syndromes: physiopathology and treatment stratagems. Can J Kidney Health Dis. 2015;2:41.

84. Kingma JG, Simard D, Rouleau JR, Drolet B, Simard C. The physiopathology of cardiorenal syndrome: a review of the potential contributions of inflammation. J Cardiovasc Dev Dis. 2017;4:21.

85. McCullough PA, Kellum JA, Haase M, Muller C, Damman K, Murray PT, et al. Pathophysiology of the cardiorenal syndromes: executive summary from the eleventh consensus conference of the Acute Dialysis Quality Initiative (ADQI). Contrib Nephrol. 2013;182:82-98.

86. Peesapati VSR, Sadik M, Verma S, Attallah MA, Khan S. Panoramic Dominance of the Immune System in Cardiorenal Syndrome Type I. Cureus. 2020;12:e9869.

87. Schefold JC, Filippatos G, Hasenfuss G, Anker SD, von Haehling S. Heart failure and kidney dysfunction: epidemiology, mechanisms and management. Nat Rev Nephrol. 2016;12:610-23.

88. Shi S, Zhang B, Li Y, Xu X, Lv J, Jia Q, et al. Mitochondrial Dysfunction: An Emerging Link in the Pathophysiology of Cardiorenal Syndrome. Front Cardiovasc Med. 2022;9:837270.

89. Virzi GM, Clementi A, Battaglia GG, Ronco C. Multi-omics approach: new potential key mechanisms implicated in cardiorenal syndromes. Cardiorenal Med. 2019;9:201-11.

90. Virzi GM, Clementi A, Brocca A, de Cal M, Vescovo G, Granata A, et al. The hemodynamic and nonhemodynamic crosstalk in cardiorenal syndrome type 1. Cardiorenal Med. 2014;4:103-12.

91. Virzi GM, Torregrossa R, Cruz DN, Chionh CY, de Cal M, Soni SS, et al. Cardiorenal Syndrome Type 1 May Be Immunologically Mediated: A Pilot Evaluation of Monocyte Apoptosis. Cardiorenal Med. 2012;2:33-42.

92. El Hadi H, Di Vincenzo A, Vettor R, Rossato M. Relationship between heart disease and liver disease: a two-way street. Cells. 2020;9:567.

93. Dos Santos ARDO, De Oliveira Zanuso B, Miola VFB, Barbalho SM, Bueno PCS, Flato UAP, et al. Adipokines, myokines, and hepatokines: crosstalk and metabolic repercussions. Int J Mol Sci. 2021;22:2639.

94. Gonzalez-Gil AM, Elizondo-Montemayor L. The role of exercise in the interplay between myokines, hepatokines, osteokines, adipokines, and modulation of inflammation for energy substrate redistribution and fat mass loss: a review. Nutrients. 2020;12:1899.

95. Oishi Y, Manabe I. Organ System Crosstalk in Cardiometabolic Disease in the Age of Multimorbidity. Front Cardiovasc Med. 2020;7:64.

96. Wiernsperger N. Hepatic function and the cardiometabolic syndrome. Diabetes Metab Syndr Obes. 2013;6:379-88.

97. Bu J, Wang Z. Cross-Talk between Gut Microbiota and Heart via the Routes of Metabolite and Immunity. Gastroenterol Res Pract. 2018;2018:6458094.

98. Collins S. A heart-adipose tissue connection in the regulation of energy metabolism. Nat Rev Endocrinol. 2014;10:157-63.

99. Ferrero KM, Koch WJ. Metabolic crosstalk between the heart and fat. Korean Circ J. 2020;50:379-94.

100. Gaborit B, Abdesselam I, Dutour A. Epicardial fat: more than just an "epi" phenomenon? Horm Metab Res. 2013;45:991-1001.

101. Jahng JW, Song E, Sweeney G. Crosstalk between the heart and peripheral organs in heart failure. Exp Mol Med. 2016;48:e217.

102. Romacho T, Elsen M, Rohrborn D, Eckel J. Adipose tissue and its role in organ crosstalk. Acta Physiol (Oxf). 2014;210:733-53.

103. Zhao S, Kusminski CM, Scherer PE. Adiponectin, leptin and cardiovascular disorders. Circ Res. 2021;128:136-49.

104. Costa C, Sampaio-Maia B, Araujo R, Nascimento DS, Ferreira-Gomes J, Pestana M, et al. Gut microbiome and organ fibrosis. Nutrients. 2022;14:352.

105. Xiong P, Zhang F, Liu F, Zhao J, Huang X, Luo D, et al. Metaflammation in glucolipid metabolic disorders: Pathogenesis and treatment. Biomed Pharmacother. 2023;161:114545.

106. Van Linthout S, Tschope C. Inflammation - cause or consequence of heart failure or both? Curr Heart Fail Rep. 2017;14:251-65.

107. Nicin L, Wagner JUG, Luxan G, Dimmeler S. Fibroblast-mediated intercellular crosstalk in the healthy and diseased heart. FEBS Lett. 2022;596:638-54.

108. Majnaric LT, Bosnic Z, Stefanic M, Wittlinger T. Cross-talk between the cytokine IL-37 and thyroid hormones in modulating chronic inflammation associated with target organ damage in age-related metabolic and vascular conditions. Int J Mol Sci. 2022;23:6456.

109. Mace ML, Egstrand S, Morevati M, Olgaard K, Lewin E. New insights to the crosstalk between vascular and bone tissue in chronic kidney disease-mineral and bone disorder. Metabolites. 2021;11:849.

110. Burtscher J, Soltany A, Visavadiya NP, Burtscher M, Millet GP, Khoramipour K, et al. Mitochondrial stress and mitokines in aging. Aging Cell. 2023;22:e13770.

111. Severinsen MCK, Pedersen BK. Muscle-Organ Crosstalk: The Emerging Roles of Myokines. Endocr Rev. 2020;41:594-609.

112. Barros D, Marques EA, Magalhaes J, Carvalho J. Energy metabolism and frailty: The potential role of exercise-induced myokines - A narrative review. Ageing Res Rev. 2022;82:101780.

113. De Nicolo B, Cataldi-Stagetti E, Diquigiovanni C, Bonora E. Calcium and reactive oxygen species signaling interplays in cardiac physiology and pathologies. Antioxidants (Basel). 2023;12:353.

114. Graf C, Ferrari N. Metabolic health-the role of adipo-myokines. Int J Mol Sci. 2019;20:6159.

115. Kiran S, Kumar V, Kumar S, Price RL, Singh UP. Adipocyte, immune cells, and miRNA crosstalk: a novel regulator of metabolic dysfunction and obesity. Cells. 2021;10:1004.

116. Wu YY, Shan SK, Lin X, Xu F, Zhong JY, Wu F, et al. Cellular Crosstalk in the Vascular Wall Microenvironment: The Role of Exosomes in Vascular Calcification. Front Cardiovasc Med. 2022;9:912358.

117. Lowenstein J, Nigam SK. Uremic Toxins in Organ Crosstalk. Front Med (Lausanne). 2021;8:592602.

118. White LE, Chaudhary R, Moore LJ, Moore FA, Hassoun HT. Surgical sepsis and organ crosstalk: the role of the kidney. J Surg Res. 2011;167:306-15.

119. Gluhovschi G, Petrică L, Sporea I, Timar R, Curescu M, Velciov S, et al. Chronic kidney disease--chronic liver disease. An immunologic cross-talk. Rom J Intern Med. 2015;53:3-12.

120. Capalbo O, Giuliani S, Ferrero-Fernandez A, Casciato P, Musso CG. Kidney-liver pathophysiological crosstalk: its characteristics and importance. Int Urol Nephrol. 2019;51:2203-7.

121. Duvigneau JC, Luis A, Gorman AM, Samali A, Kaltenecker D, Moriggl R, et al. Crosstalk between inflammatory mediators and endoplasmic reticulum stress in liver diseases. Cytokine. 2019;124:154577.

122. Lane K, Dixon JJ, MacPhee IA, Philips BJ. Renohepatic crosstalk: does acute kidney injury cause liver dysfunction? Nephrol Dial Transplant. 2013;28:1634-47.

123. Capalbo O, Giuliani S, Ferrero-Fernandez A, Casciato P, Musso CG. Kidney-liver pathophysiological crosstalk: its characteristics and importance. Int Urol Nephrol. 2019;51:2203-2207.

124. Colombo I, Aiello-Battan F, Elena R, Ruiz A, Petraglia L, Musso CG. Kidney-gut crosstalk in renal disease. Ir J Med Sci. 2021;190:1205-12.

125. Karava V, Christoforidis A, Kondou A, Dotis J, Printza N. Update on the Crosstalk Between Adipose Tissue and Mineral Balance in General Population and Chronic Kidney Disease. Front Pediatr. 2021;9:696942.

126. Zhu Q, Scherer PE. Immunologic and endocrine functions of adipose tissue: implications for kidney disease. Nat Rev Nephrol. 2018;14:105-20.

127. Choi HW, Lee KW, Kim YH. Microbiome in urological diseases: axis crosstalk and bladder disorders. Investig Clin Urol. 2023;64:126-39.

128. Virzì G, Day S, De Cal M, Vescovo G, Ronco C. Heart-kidney crosstalk and role of humoral signaling in critical illness. Crit Care. 2014;18:201.

129. Andrade-Oliveira V, Foresto-Neto O, Watanabe IKM, Zatz R, Camara NOS. Inflammation in Renal Diseases: New and Old Players. Front Pharmacol. 2019;10:1192.

130. Cabarcas-Barbosa O, Capalbo O, Ferrero-Fernandez A, Musso CG. Kidney-placenta crosstalk in health and disease. Clin Kidney J. 2022;15:1284-9.

131. Grange C, Bussolati B. Extracellular vesicles in kidney disease. Nat Rev Nephrol. 2022;18:499-513.

132. Messerer DAC, Halbgebauer R, Nilsson B, Pavenstadt H, Radermacher P, Huber-Lang M. Immunopathophysiology of trauma-related acute kidney injury. Nat Rev Nephrol. 2021;17:91-111.

133. Dousdampanis P, Trigka K, Vagenakis GA, Fourtounas C. The thyroid and the kidney: a complex interplay in health and disease. Int J Artif Organs. 2014;37:1-12.

134. Ohlendieck K, Swandulla D. Complexity of skeletal muscle degeneration: multi-systems pathophysiology and organ crosstalk in dystrophinopathy. Pflugers Arch. 2021;473:1813-39.

135. Zhou S, Cheing GLY, Cheung AKK. Role of exosomes and exosomal microRNA in muscle-Kidney crosstalk in chronic kidney disease. Front Cell Dev Biol. 2022;10:951837.

136. de Jong PR, Gonzalez-Navajas JM, Jansen NJ. The digestive tract as the origin of systemic inflammation. Crit Care. 2016;20:279.

137. Bauer M. The liver-gut-axis: initiator and responder to sepsis. Curr Opin Crit Care. 2022;28:216-20.

138. Albuquerque-Souza E, Sahingur SE. Periodontitis, chronic liver diseases, and the emerging oral-gut-liver axis. Periodontol 2000. 2022;89:125-41.

139. Di Ciaula A, Baj J, Garruti G, Celano G, De Angelis M, Wang HH, et al. Liver steatosis, gut-liver axis, microbiome and environmental factors. A never-ending bidirectional cross-talk. J Clin Med. 2020;9:2648.

140. Li X, Wang H. Multiple organs involved in the pathogenesis of non-alcoholic fatty liver disease. Cell Biosci. 2020;10:140.

141. Wahlstrom A, Sayin SI, Marschall HU, Backhed F. Intestinal Crosstalk between Bile Acids and Microbiota and Its Impact on Host Metabolism. Cell Metab. 2016;24:41-50.

142. Zhang S, Lu S, Li Z. Extrahepatic factors in hepatic immune regulation. Front Immunol. 2022;13:941721.

143. Zhang X, Ji X, Wang Q, Li JZ. New insight into inter-organ crosstalk contributing to the pathogenesis of non-alcoholic fatty liver disease (NAFLD). Protein Cell. 2018;9:164-77.

144. Di Ciaula A, Baj J, Garruti G, Celano G, De Angelis M, Wang HH, et al. Liver Steatosis, Gut-Liver Axis, Microbiome and Environmental Factors. A Never-Ending Bidirectional Cross-Talk. J Clin Med. 2020;9.

145. Gerner RR, Wieser V, Moschen AR, Tilg H. Metabolic inflammation: role of cytokines in the crosstalk between adipose tissue and liver. Can J Physiol Pharmacol. 2013;91:867-72.

146. Huang Z, Xu A. Adipose Extracellular Vesicles in Intercellular and Inter-Organ Crosstalk in Metabolic Health and Diseases. Front Immunol. 2021;12:608680.

147. Huh JY, Park YJ, Ham M, Kim JB. Crosstalk between adipocytes and immune cells in adipose tissue inflammation and metabolic dysregulation in obesity. Mol Cells. 2014;37:365-71.

148. Ji C, Guo X. The clinical potential of circulating microRNAs in obesity. Nat Rev Endocrinol. 2019;15:731-43.

149. Ma M, Duan R, Zhong H, Liang T, Guo L. The Crosstalk between Fat Homeostasis and Liver Regional Immunity in NAFLD. J Immunol Res. 2019;2019:3954890.

150. Nov O, Shapiro H, Ovadia H, Tarnovscki T, Dvir I, Shemesh E, et al. Interleukin-1beta regulates fat-liver crosstalk in obesity by auto-paracrine modulation of adipose tissue inflammation and expandability. PLoS One. 2013;8:e53626.

151. Oh KJ, Lee DS, Kim WK, Han BS, Lee SC, Bae KH. Metabolic adaptation in obesity and type II diabetes: myokines, adipokines and hepatokines. Int J Mol Sci. 2016;18:8.

152. van den Hoek AM, de Jong J, Worms N, van Nieuwkoop A, Voskuilen M, Menke AL, et al. Diet and exercise reduce pre-existing NASH and fibrosis and have additional beneficial effects on the vasculature, adipose tissue and skeletal muscle via organ-crosstalk. Metabolism. 2021;124:154873.

153. Ye DW, Rong XL, Xu AM, Guo J. Liver-adipose tissue crosstalk: a key player in the pathogenesis of glucolipid metabolic disease. Chin J Integr Med. 2017;23:410-4.

154. Wu L, Li J, Feng J, Ji J, Yu Q, Li Y, et al. Crosstalk between PPARs and gut microbiota in NAFLD. Biomed Pharmacother. 2021;136:111255.

155. Li Y, Palmer A, Lupu L, Huber-Lang M. Inflammatory response to the ischaemia-reperfusion insult in the liver after major tissue trauma. Eur J Trauma Emerg Surg. 2022;48:4431-44.

156. Lopez-Bermudo L, Luque-Sierra A, Maya-Miles D, Gallego-Duran R, Ampuero J, Romero-Gomez M, et al. Contribution of Liver and Pancreatic Islet Crosstalk to beta-Cell Function/Dysfunction in the Presence of Fatty Liver. Front Endocrinol (Lausanne). 2022;13:892672.

157. Manieri E, Sabio G. Stress kinases in the modulation of metabolism and energy balance. J Mol Endocrinol. 2015;55:R11-22.

158. Von-Hafe M, Borges-Canha M, Vale C, Leite AR, Neves JS, Carvalho D, et al. Nonalcoholic fatty liver disease and endocrine axes-a scoping review. Metabolites. 2022;12:298.

159. Chakravarthy MV, Siddiqui MS, Forsgren MF, Sanyal AJ. Harnessing Muscle-Liver Crosstalk to Treat Nonalcoholic Steatohepatitis. Front Endocrinol (Lausanne). 2020;11:592373.

160. Kim KH, Lee MS. Autophagy as a crosstalk mediator of metabolic organs in regulation of energy metabolism. Rev Endocr Metab Disord. 2014;15:11-20.

161. Maurer J, Hoene M, Weigert C. Signals from the circle: tricarboxylic acid cycle intermediates as myometabokines. Metabolites. 2021;11:474.

162. Pasmans K, Adriaens ME, Olinga P, Langen R, Rensen SS, Schaap FG, et al. Hepatic steatosis contributes to the development of muscle atrophy via inter-organ crosstalk. Front Endocrinol (Lausanne). 2021;12:733625.

163. Renzini A, D'Onghia M, Coletti D, Moresi V. Histone Deacetylases as Modulators of the Crosstalk Between Skeletal Muscle and Other Organs. Front Physiol. 2022;13:706003.

164. Bleau C, Karelis AD, St-Pierre DH, Lamontagne L. Crosstalk between intestinal microbiota, adipose tissue and skeletal muscle as an early event in systemic low-grade inflammation and the development of obesity and diabetes. Diabetes Metab Res Rev. 2015;31:545-61.

165. Geurts L, Neyrinck AM, Delzenne NM, Knauf C, Cani PD. Gut microbiota controls adipose tissue expansion, gut barrier and glucose metabolism: novel insights into molecular targets and interventions using prebiotics. Benef Microbes. 2014;5:3-17.

166. May KS, Den Hartigh LJ. Gut microbial-derived short chain fatty acids: impact on adipose tissue physiology. Nutrients. 2023;15:272.

167. Rodriguez J, Delzenne NM. Modulation of the gut microbiota-adipose tissue-muscle interactions by prebiotics. J Endocrinol. 2021;249:R1-23.

168. Rosendo-Silva D, Matafome P. Gut-adipose tissue crosstalk: A bridge to novel therapeutic targets in metabolic syndrome? Obes Rev. 2021;22:e13130.

169. Vajro P, Paolella G, Fasano A. Microbiota and gut-liver axis: their influences on obesity and obesity-related liver disease. J Pediatr Gastroenterol Nutr. 2013;56:461-8.

170. Zhang Y, Chen R, Zhang D, Qi S, Liu Y. Metabolite interactions between host and microbiota during health and disease: Which feeds the other? Biomed Pharmacother. 2023;160:114295.

171. Corriero A, Gadaleta RM, Puntillo F, Inchingolo F, Moschetta A, Brienza N. The central role of the gut in intensive care. Crit Care. 2022;26:379.

172. El Aidy S, Dinan TG, Cryan JF. Gut Microbiota: The Conductor in the Orchestra of Immune-Neuroendocrine Communication. Clin Ther. 2015;37:954-67.

173. Fernandez-Veledo S, Vendrell J. Gut microbiota-derived succinate: friend or foe in human metabolic diseases? Rev Endocr Metab Disord. 2019;20:439-47.

174. Krautkramer KA, Fan J, Backhed F. Gut microbial metabolites as multi-kingdom intermediates. Nat Rev Microbiol. 2021;19:77-94.

175. Assis V, De Sousa Neto IV, Ribeiro FM, De Cassia Marqueti R, Franco OL, Da Silva Aguiar S, et al. The emerging role of the aging process and exercise training on the crosstalk between gut microbiota and telomere length. Int J Environ Res Public Health. 2022;19:7810.

176. Cong J, Zhou P, Zhang R. Intestinal microbiota-derived short chain fatty acids in host health and disease. Nutrients. 2022;14:1977.

177. Fernandez-Millan E, Guillen C. Multi-organ crosstalk with endocrine pancreas: a focus on how gut microbiota shapes pancreatic beta-cells. Biomolecules. 2022;12:104.

178. Shim JA, Ryu JH, Jo Y, Hong C. The role of gut microbiota in T cell immunity and immune mediated disorders. Int J Biol Sci. 2023;19:1178-91.

179. Zhang Z, Tanaka I, Pan Z, Ernst PB, Kiyono H, Kurashima Y. Intestinal homeostasis and inflammation: gut microbiota at the crossroads of pancreas-intestinal barrier axis. Eur J Immunol. 2022;52:1035-46.

180. Zhou A, Yuan Y, Yang M, Huang Y, Li X, Li S, et al. Crosstalk Between the Gut Microbiota and Epithelial Cells Under Physiological and Infectious Conditions. Front Cell Infect Microbiol. 2022;12:832672.

181. Chawla LS, Fink M, Goldstein SL, Opal S, Gomez A, Murray P, et al. The epithelium as a target in sepsis. Shock. 2016;45:249-58.

182. Greer RL, Morgun A, Shulzhenko N. Bridging immunity and lipid metabolism by gut microbiota. J Allergy Clin Immunol. 2013;132:253-62; quiz 263.

183. Park EJ, Shimaoka M, Kiyono H. Functional Flexibility of Exosomes and MicroRNAs of Intestinal Epithelial Cells in Affecting Inflammation. Front Mol Biosci. 2022;9:854487.

184. Sanwlani R, Fonseka P, Chitti SV, Mathivanan S. Milk-derived extracellular vesicles in inter-organism, cross-species communication and drug delivery. Proteomes. 2020;8:11.

185. Teunis C, Nieuwdorp M, Hanssen N. Interactions between Tryptophan Metabolism, the Gut Microbiome and the Immune System as Potential Drivers of Non-Alcoholic Fatty Liver Disease (NAFLD) and Metabolic Diseases. Metabolites. 2022;12.

186. Takayanagi H. Osteoimmunology - bidirectional dialogue and inevitable union of the fields of bone and immunity. Proc Jpn Acad Ser B Phys Biol Sci. 2020;96:159-69.

187. Gizard F, Fernandez A, De Vadder F. Interactions between gut microbiota and skeletal muscle. Nutr Metab Insights. 2020;13:1178638820980490.

188. Frampton J, Murphy KG, Frost G, Chambers ES. Short-chain fatty acids as potential regulators of skeletal muscle metabolism and function. Nat Metab. 2020;2:840-8.

189. Rakib A, Kiran S, Mandal M, Singh UP. MicroRNAs: a crossroad that connects obesity to immunity and aging. Immun Ageing. 2022;19:64.

190. Tanaka M. Molecular mechanism of obesity-induced adipose tissue inflammation; the role of mincle in adipose tissue fibrosis and ectopic lipid accumulation. Endocr J. 2020;67:107-11.

191. Armutcu F. Organ crosstalk: the potent roles of inflammation and fibrotic changes in the course of organ interactions. Inflamm Res. 2019;68:825-39.

192. Thomas SS, Mitch WE. Parathyroid hormone stimulates adipose tissue browning: a pathway to muscle wasting. Curr Opin Clin Nutr Metab Care. 2017;20:153-7.

193. Kirk B, Feehan J, Lombardi G, Duque G. Muscle, Bone, and Fat Crosstalk: the Biological Role of Myokines, Osteokines, and Adipokines. Curr Osteoporos Rep. 2020;18:388-400.

194. Gomez MPA, Benavent CA, Simoni P, Aparisi F, Guglielmi G, Bazzocchi A. Fat and bone: the multiperspective analysis of a close relationship. Quant Imaging Med Surg. 2020;10:1614-35.

195. Coles CA. Adipokines in Healthy Skeletal Muscle and Metabolic Disease. Adv Exp Med Biol. 2016;900:133-60.

196. Jiang S, Bae JH, Wang Y, Song W. The potential roles of myokines in adipose tissue metabolism with exercise and cold exposure. Int J Mol Sci. 2022;23:11523.

197. Li F, Li Y, Duan Y, Hu CA, Tang Y, Yin Y. Myokines and adipokines: Involvement in the crosstalk between skeletal muscle and adipose tissue. Cytokine Growth Factor Rev. 2017;33:73-82.

198. Rome S. Muscle and adipose tissue communicate with extracellular vesicles. Int J Mol Sci. 2022;23:7052.

199. Wang YC, Li Y, Wang XY, Zhang D, Zhang H, Wu Q, et al. Circulating miR-130b mediates metabolic crosstalk between fat and muscle in overweight/obesity. Diabetologia. 2013;56:2275-85.

200. Yue B, Wang H, Cai X, Wang J, Chai Z, Peng W, et al. Adipose-secreted exosomes and their pathophysiologic effects on skeletal muscle. Int J Mol Sci. 2022;23:12411.

201. Christ A, Lauterbach M, Latz E. Western Diet and the Immune System: An Inflammatory Connection. Immunity. 2019;51:794-811.

202. Shirakawa J, De Jesus DF, Kulkarni RN. Exploring inter-organ crosstalk to uncover mechanisms that regulate beta-cell function and mass. Eur J Clin Nutr. 2017;71:896-903.

203. Eisele PS, Handschin C. Functional crosstalk of PGC-1 coactivators and inflammation in skeletal muscle pathophysiology. Semin Immunopathol. 2014;36:27-53.

204. Rutti S, Dusaulcy R, Hansen JS, Howald C, Dermitzakis ET, Pedersen BK, et al. Angiogenin and Osteoprotegerin are type II muscle specific myokines protecting pancreatic beta-cells against proinflammatory cytokines. Sci Rep. 2018;8:10072.

205. Rogeri PS, Gasparini SO, Martins GL, Costa LKF, Araujo CC, Lugaresi R, et al. Crosstalk Between Skeletal Muscle and Immune System: Which Roles Do IL-6 and Glutamine Play? Front Physiol. 2020;11:582258.

206. Brotto M, Johnson ML. Endocrine crosstalk between muscle and bone. Curr Osteoporos Rep. 2014;12:135-41.

207. Karstoft K, Pedersen BK. Skeletal muscle as a gene regulatory endocrine organ. Curr Opin Clin Nutr Metab Care. 2016;19:270-5.

208. Mizgier ML, Casas M, Contreras-Ferrat A, Llanos P, Galgani JE. Potential role of skeletal muscle glucose metabolism on the regulation of insulin secretion. Obes Rev. 2014;15:587-97.

209. Mizgier ML, Fernandez-Verdejo R, Cherfan J, Pinget M, Bouzakri K, Galgani JE. Insights on the Role of Putative Muscle-Derived Factors on Pancreatic Beta Cell Function. Front Physiol. 2019;10:1024.

210. Bonewald L. Use it or lose it to age: a review of bone and muscle communication. Bone. 2019;120:212-8.

211. Cariati I, Bonanni R, Onorato F, Mastrogregori A, Rossi D, Iundusi R, et al. Role of physical activity in bone-muscle crosstalk: biological aspects and clinical implications. J Funct Morphol Kinesiol. 2021;6:55.

212. Cianferotti L, Brandi ML. Muscle-bone interactions: basic and clinical aspects. Endocrine. 2014;45:165-77.

213. Hamrick MW. Role of the Cytokine-like Hormone Leptin in Muscle-bone Crosstalk with Aging. J Bone Metab. 2017;24:1-8.

214. He C, He W, Hou J, Chen K, Huang M, Yang M, et al. Bone and Muscle Crosstalk in Aging. Front Cell Dev Biol. 2020;8:585644.

215. Karsenty G, Mera P. Molecular bases of the crosstalk between bone and muscle. Bone. 2018;115:43-9.

216. Leal DV, Ferreira A, Watson EL, Wilund KR, Viana JL. Muscle-bone crosstalk in chronic kidney disease: the potential modulatory effects of exercise. Calcif Tissue Int. 2021;108:461-75.

217. Li G, Zhang L, Wang D, AI Qudsy L, Jiang JX, Xu H, et al. Muscle-bone crosstalk and potential therapies for sarco-osteoporosis. J Cell Biochem. 2019;120:14262-73.
